# Cadmium Stress Signaling Pathways in Plants: Molecular Responses and Mechanisms

**DOI:** 10.3390/cimb46060361

**Published:** 2024-06-14

**Authors:** Valentina Vitelli, Agnese Giamborino, Andrea Bertolini, Alessandro Saba, Andrea Andreucci

**Affiliations:** 1 Department of Biology, University of Pisa, 56126 Pisa, Italy; v.vitelli@student.unisi.it; 2 Department of Surgical, Medical and Molecular Pathology and Critical Care Area, University of Pisa, 56126 Pisa, Italy; a.giamborino@student.unisi.it (A.G.); a.bertolini2@student.unisi.it (A.B.); alessandro.saba@unipi.it (A.S.)

**Keywords:** pollution, heavy metal, cadmium, phytochelatins, glutathione, transporter

## Abstract

Heavy metal (HM) pollution, specifically cadmium (Cd) contamination, is a worldwide concern for its consequences for plant health and ecosystem stability. This review sheds light on the intricate mechanisms underlying Cd toxicity in plants and the various strategies employed by these organisms to mitigate its adverse effects. From molecular responses to physiological adaptations, plants have evolved sophisticated defense mechanisms to counteract Cd stress. We highlighted the role of phytochelatins (PC_n_) in plant detoxification, which chelate and sequester Cd ions to prevent their accumulation and minimize toxicity. Additionally, we explored the involvement of glutathione (GSH) in mitigating oxidative damage caused by Cd exposure and discussed the regulatory mechanisms governing GSH biosynthesis. We highlighted the role of transporter proteins, such as ATP-binding cassette transporters (ABCs) and heavy metal ATPases (HMAs), in mediating the uptake, sequestration, and detoxification of Cd in plants. Overall, this work offered valuable insights into the physiological, molecular, and biochemical mechanisms underlying plant responses to Cd stress, providing a basis for strategies to alleviate the unfavorable effects of HM pollution on plant health and ecosystem resilience.

## 1. Introduction: Environmental Heavy Metal Contamination

Heavy metals (HMs) are elements characterized by an atomic number greater than 20, an atomic density exceeding 5 g/cm^3^, and metallic properties. Transition metals, like cadmium (Cd), mercury (Hg), and chromium (Cr), elements in the bottom left section of the periodic table like lead (Pb) and tin (Sn), and certain metalloids like arsenic (As) are essentially what are referred to as HMs. The latter can be broadly classified into two categories: essential and non-essential HMs. The first ones are necessary for living organisms to perform fundamental processes such as growth, metabolism, and organ development. Therefore, HMs, such as iron (Fe), zinc (Zn), copper (Cu), and cobalt (Co) function as cofactors that support redox and protein structure processes, making them crucial for plant growth and development. The non-essential ones are dangerous because they can bind to a variety of functional groups in biomolecules, including amino, carboxylic acid, and sulfur-containing groups [1]. Moreover, they compete with the plant for nutrient uptake; they are dangerous even at low concentrations as they can result in macronutrient deficiencies. Increased HM buildup results in acute toxicity, growth inhibition, and ultimately plant death [2]. In contrast to organic pollutants, HMs are non-biodegradable and tend to accumulate in living organisms [3]. The pervasive problem of HM pollution in the environment is a significant risk to public health worldwide. Reports indicate that 20 million hectares of China’s farmland, constituting roughly 20% of the nation’s total agricultural area, are affected by HM pollution, notably Cd, Pb, As, and Hg. Within this affected area, 14,000 hectares are particularly afflicted by Cd contamination [1,4]. Groundwater, agricultural products, and crops alike will soon suffer the far-reaching consequences of the presence of Cd in soil [5]. Pollution of the environment by HMs can result from both natural processes and human activities. Among the latter, the primary artificial causes of soil contamination include farming practices, urban sprawl, industrial activities, mining operations [6], and the expansion of transport infrastructure [7]. Industrial operations contribute to ecological pollution since HMs and their numerous chemical combinations are frequently employed as catalysts in chemical reactions. Transportation-wise, the emission of gases from tires, brakes, and gasoline fuel have been shown to be the main contributors of Pb and Cd emissions to the air due to increased traffic [8]. Concerning the mining activity, although there are no mines present nearby, agricultural goods cultivated in roadside fields have higher levels of HM contamination than crops grown in other parts [9]. Moreover, the mine environment is further polluted by soil-borne metal particles carried by wind or mineral transportation [10]. The soil pollution of HMs in close proximity to mines is also significantly influenced by climate [10,11]. The expanding trash mounds have a negative effect on the surrounding areas of mining and could potentially transform some agricultural regions into wasteland [12]. Besides, one of the primary causes of ecological contamination in these areas is the water utilized in the mining process. People living close to mines suffer from a variety of autoimmune and non-autoimmune disorders that can be fatal due to toxic HMs in the surrounding soil [12]. Furthermore, human-induced soil modifications, such as the standard agricultural methods used in different areas, cause crops to take in HMs. The most common causes of liquid-induced HM environmental pollution are the application of phosphate fertilizers and the irrigation of agricultural lands with dirty water [13].

One trace element that is not necessary for biological systems yet is present in large quantities in the environment is Cd. Due to its exceptional toxicity to all biological systems, Cd is an HM that is frequently released into the environment by both natural and man-made sources [14]. For these reasons, it is considered a major metal pollutant [15,16]. Cd and its compounds are soluble, making them more mobile than other metals in environmental matrices. They also have high bioavailability, which makes them easily absorbable by plants. Once inside the plant, Cd can interfere with important physiological processes [17,18]. Given that Cd is highly toxic, this poses a significant risk to human health, leading to cancer and extensive environmental harm [19]. According to numerous studies, dumps often contain Cd levels exceeding the permissible limit in soil [20,21,22,23]. With a global mean of 0.36 mg/kg, Cd usually occurs in soils at amounts ranging from 0.01 to 1 mg/kg [16] and can be found in soil water at up to 5 μg/L [24] and groundwater at up to 1 μg/L [25]. Increases in Cd concentrations in soils and groundwater, which are crucial for preserving a wholesome food supply and clean drinking water, can come from both natural and man-made sources. The WHO Guidelines for Drinking-Water Quality recommend a maximum value of 3 μg/L for Cd. The potential for Cd accumulation in plants to transfer to humans via the food chain, causing serious harm, is nowadays a considerable concern [26,27]. Due to competition with calcium (Ca) and other nutrients, chronic Cd poisoning, also known as itai-itai disease and initially identified in Japan in the early 20th century, results in renal tubular dysfunction, osteomalacia, and osteoporosis [28,29,30]. According to Khan et al. 2017 [30], Cd exposure is also linked to problems with glucose metabolism, lung and breast cancer, myocardial infarction, and heart failure.

## 2. Effects of Cd in Plants

Crops, agricultural goods, and groundwater are significantly affected by Cd [5], a trace element that is not necessary for plant growth, although it does accumulate in all sections of plants after absorption [2,31]. The first signs of Cd toxic effects on plants include browning of the roots, a decrease in the number of lateral roots, and the death of the tips of the roots due to inhibition of root growth. Furthermore, the accumulation of Cd could result in stem development retardation, terminal leaf chlorosis, and leaf curl [32,33]. By substituting the sulfhydryl groups of proteins and enzymes, Cd replaces the essential elements of enzyme active sites needed in biological reactions. It also modifies macromolecule conformation, causes protein denaturation, and deteriorates cell membranes. The plant defense system reacts to the entry of Cd into plant cells by accumulating proline [34,35,36], variations in the synthesis and levels of phytohormones [37,38,39], and the initiation of several signaling pathways, including the Ca-calmodulin pathway as well as the activation of various antioxidant enzymes [40,41].

**Figure 1:**
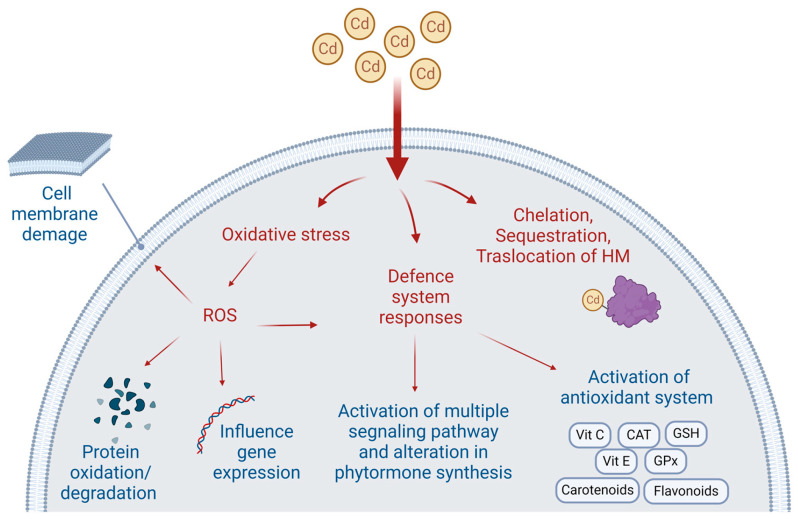
Toxic effects of Cd in plants. Induction of oxidative stress leads to ROS generation, resulting in cell membrane damage, protein oxidation, and degradation, with effects on gene expression. Cellular responses to Cd entry include activation of the defense system as well as chelation, sequestration, and translocation of the HM.

Cd significantly increases the production of reactive oxygen species (ROS) after binding with proteins and enzyme complexes, which leads to oxidative stress [42,43]. Reactive nitrogen species (RNS) and ROS are byproducts of regular cellular metabolism and are known to play two distinct roles in biological systems [44]. High ROS levels cause the cell to arrest and eventually become necrotic or apoptotic, whereas low ROS levels accelerate the cell cycle’s progression [45]. The toxicity due to elevated ROS concentrations reflects the oxidation of proteins, DNA, and membrane lipids, as well as the breakdown of polyphenols, catecholamines, and thiols. Previous reports highlight ROS dual function in plant defense systems as a signaling molecule and as a source of toxicity [46,47]. According to multiple studies [48,49] these molecules are produced by the Fenton or Haber-Weiss reaction, respiratory burst oxidase homologs (also known as NADPH oxidases), and the inhibition of antioxidative enzymes, such as oxidases and peroxidases. The cell has evolved a sophisticated antioxidant system that may shield biological molecules from potentially hazardous, purely physiological, species-producing oxidative stress through both enzymatic and non-enzymatic methods. Proteins like catalase (CAT), glutathione peroxidase (GPx), ascorbic acid (Vitamin C), ϱ-tocopherol (Vitamin E), glutathione (GSH), carotenoids, flavonoids, and other antioxidant agents are part of the enzymatic defense system [50] (Figure 1).

## 3. Plant Responses to Cd Stress and Detoxification via PC_n_ Production

Different plant species are affected by nutrient variance under Cd stress [15,51,52]. Certain molecular alterations control Cd mitigation, tolerance, and adaptation, including the overexpression of specific genes. It has been observed that natural resistance and macrophage proteins (NRAMPs), iron-regulated transporter-like proteins (IRTs), zinc-regulated transporter-like proteins (ZIPs), heavy metal ATPases (HMAs), and metal tolerance or transporter proteins (MTPs) play roles in the uptake, translocation, and sequestration of Cd within plants [53]. Jia et al. 2019 [54] revealed that a high concentration of Cd (6.5 µM Cd) increases the expression of genes (*SlQUA1* and *SlPME1*) associated with pectin synthesis in the cell walls of tomatoes, leading to an increase in pectin methyl esterase activity and ion binding sites, which counteracts Cd stress. Additionally, Song, Feng, et al. 2017 [55] demonstrated that overexpression of *AtFC1* in *A. thaliana* enhances Cd accumulation and tolerance under Cd exposure. Conversely, genes like *HvIRT1* and *HvNramp5* in barley [56] downregulate Cd uptake, while lower expression of *HMA-A*, *HMA-B*, *IRT1*, and *ZIP1* in tobacco leads to reduced Cd accumulation [57]. Genes such as *ZIP2*, *ZIP3*, *IRT1*, *HMA2*, and *HMA4* and *CAX4*, *HMA3*, *MRP7*, *MTP3*, and *COPT5* are overexpressed in Pak choi (*B. rapa*) grown in sand artificially contaminated with 10 mg Cd Kg^−1^ [58]. Other studies on the response to Cd stress have been conducted in rape, *A. thaliana*, maize, rice, and other crops [59,60]. *NcNramp1* was shown to be crucial for the process of plant absorption and accumulation of the ionic form Cd^2+^ when transcriptome technology was used to examine *N. caerulescens* under Cd stress in various genotypes [61].

As a result of the significant toxic impact on plant growth and development, plants have evolved a variety of tolerance and detoxification strategies to control the uptake, transportation, and accumulation of Cd and lessen its toxicity [62]. The plant root hairs, along with the cuticle and epidermis, produce protective tissue to keep Cd out [63]. They also release organic acids, including citric, malic, and lactic acids, which mix with Cd to form compounds that prevent Cd from passing through membranes. In order to reduce the quantity of Cd that migrates from the soil into the root, they also modify the pH and EH of the rhizosphere [64]. Furthermore, to reduce the toxicity of Cd to cell activity, Cd that does enter cells will be enriched in vacuoles. This enrichment occurs due to the ability of polysaccharides and proteins in the cell wall to bond with the metal and prevent it from entering the cell [65].

**Figure 2:**
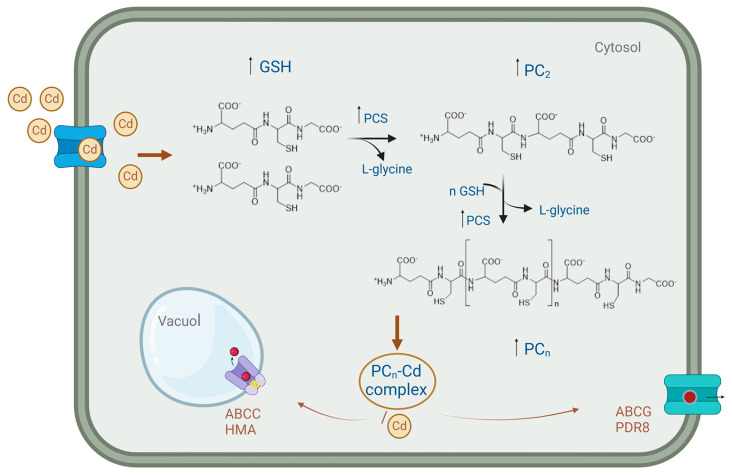
The Cd-PC_n_ complex. Cd influx into cells induces overexpression of PCS and involves glutathione in PC_2_ formation, resulting in the synthesis of other PC_n_. This production of PC_n_ leads to cadmium chelation, and the complex is transported to the vacuole for detoxification or removed from the cell via membrane transporters and efflux pumps.

Plants, particularly the hyperaccumulator ones, react in different ways to the toxicity of HMs, including Cd. HM chelation utilizing peptide ligands called phytochelatins (PC_n_) is one of the novel defense mechanisms [66]. These peptides are primarily composed of (c-Glu-Cys)n-Gly, with (2 < *n* < 11), although other PC_n_ structures such as (c-Glu-Cys)n-Glu, (c-Glu-Cys)n-Ser, and (c-Glu-Cys)n-c-Ala have also been identified [67,68]. PC_n_ are synthesized from GSH by the activation of the enzyme phytochelatin synthase (PCS), a γ-EC dipeptidyl (trans)peptidase that is constitutively expressed in the plant cytosol [69,70,71]. Synthesized PC_n_ can form a complex with Cd, referred to as a chelator-Cd complex. Intracellular Cd promotes PCS activity, and the subsequent metal-induced production of PC_n_ allows intracellular Cd detoxification [72]. Indeed, the PC_n_ complex aids in transporting Cd from the cytosol into the vacuole, where it becomes inactive [73,74] (Figure 2).

A study reports the exogenous presence of GSH to reduce Cd translocation to shoots and enhance Cd accumulation in *B. campestris* roots treated with 20 mM of Cd [40]. According to the authors, exogenous GSH promotes the synthesis of PC_n_ by increasing Cd chelation and facilitating its translocation into the root vacuoles. In fact, recent studies [72,75,76,77] confirm that exposure to Cd raises GSH levels in addition to activating the constitutively expressed enzyme PCS in *M. polymorpha* (*MpPCS*), which significantly increases the amount of PC_n_ generated overall. Particularly, a detailed gene expression analysis confirmed the constitutive transcription of *MpPCS*; indeed, Cd is able to induce the activity, but not the gene expression, of the enzyme [72]. As a result, PC_n_ synthesis is essential for plant detoxification and tolerance to high Cd concentrations. Another notable instance is seen in rice plants, where PC_n_ increases by 23% and 47%, respectively, in response to 1 and 2 mM Cd [78]. Similarly, *B. auriculata* treated with 125 mM of Cd showed similar outcomes [70]. In response to metallic micronutrients including Fe, Zn, Cu, and the hazardous element Cd, PC_n_ activation has recently been found in tracheophytes (mostly in angiosperms), as well as bryophytes and charophytes [79,80,81]. Therefore, *M. polymorpha* may be a great model organism to study the cellular and molecular mechanisms involved in Cd detoxification. Studies examining how metal detoxifying mechanisms evolved from aquatic to terrestrial environments can greatly benefit from the study of bryophytes. Not to mention, these plants do not form strong hydrophobic barriers, have a very high surface/volume ratio, an elevated cation exchange capacity, and are hence more susceptible to unrestricted, seemingly uncontrolled metal absorption [18,82]. Consequently, bryophytes have been employed as a crucial biological monitoring system for metal environmental pollution because of their widespread distribution [82,83,84]. Additionally, a study conducted by Bellini et al. 2020 [85] examined the medium acidification of this liverwort and its thiol-peptide synthesis (GSH, PC_n_) following a 72 h exposure to Cd. The loss of selective permeability, a well-known property of biological membranes, can lead to swelling or shrinking of cell compartments due to HM-induced membrane damage or energy loss. Moreover, changes in chloroplast ultrastructure have been linked to HM concentrations in studies conducted both in the field and in vitro on bryophytes [83,86]. *L. riparium* has been explored to determine whether this moss could be suitable for biomonitoring in natural field conditions. Recent studies showed that Cd exposure severely affected the amount of photosynthesis, PC_n_, GSH, and nitrogen metabolism in *L. riparium* plants. Nitrogen (N_2_) and Cd metabolism are known to be closely correlated [87,88,89,90]. N_2_ is typically associated with the abiotic stress response because it competes with essential reductants required for antioxidant responses, particularly in the presence of metals and metalloids [89,91]. High N_2_ levels promote the uptake of Fe, Zn, Cu, Ca, Hg, and other cations in certain plants [90]. Instead, the activity of glutamate dehydrogenase, another important enzyme involved in abiotic stress, is reduced because it serves as a substitute route for assimilating N and detoxifies excess NH_4_ that is produced under stressful circumstances [91,92]. On the other hand, several major crops, such as rice, beans, peas, and corn, exhibited reduced glutathione synthase (GS) activity in response to HM uptake [93,94]. Research conducted by Maresca et al., 2022 [95] demonstrated that *L. riparium* has a natural tendency to increase the occurrence of GS2 proteins. Cd exposure causes the metal to be chelated in the roots and transferred to the shoots via GSH and PC_n_ [90]. Maintaining this chelation mechanism, which controls the amount of GSH and non-protein thiols, as well as the expression of *PCS* and GSH synthase genes, requires a sufficient absorption of N_2_ [90].

## 4. Role of GSH in Mitigating Cd-Induced Abiotic Stress

Due to the implication of GSH in mitigating HM stress, which is linked to its involvement in PC_n_ synthesis, it is relevant to deepen our understanding of the GSH response during abiotic stress. Two ATP-dependent enzymes, glutamate-cysteine ligase (GSH1, previously known as γ-glutamylcysteine synthetase) and GSH synthase (GSH2), sequentially synthesize GSH from its three amino acid building blocks [96]. The first enzyme, GSH1, conjugates Cys and Glu to produce the dipeptide γ-glutamylcysteine. The second step is catalyzed by GSH2, which joins Gly to γ-glutamylcysteine to generate GSH. Growth and viability are restored when cytosol-specific GSH2 is added to the seedling-lethal GSH2 mutant strains. Therefore, for normal plant growth, cytosolic production of GSH alone is sufficient [97]. Although both *GSH1* and *GSH2* are relatively widely expressed in plant tissues, biotic stressors such as methyl jasmonate and high light [98], as well as abiotic stresses like HMs [99,100], but not H_2_O_2_ or oxidative agents [99], increase transcriptional levels of both *GSH1* and *GSH2*. Its homodimerization is therefore thought to increase enzyme stability by shielding sensitive regions [101]. Since the chloroplast is home to GSH1, the redox state of this organelle plays a crucial role in controlling the synthesis of GSH. Due to the inherent instability of oxygenic photosynthesis, chloroplasts are highly reactive to unfavorable environmental conditions, which subsequently provides quick responses to abiotic and biotic challenges [102]. The recognized function of GSH in maintaining intracellular redox homeostasis largely depends on the redox control of GSH biosynthesizing enzymes. Indeed, oxidative stress-induced metabolic circumstances create a demand for GSH and the production of its oxidated form, GSSG, while also promoting GSH synthesis through the post-translational activation of GSH1. Consequently, elevated GSH levels establish a reducing environment, triggering a regulatory feedback loop to slow down GSH synthesis and decrease GSH1 activity. The activity of the sulfate assimilation pathway also influences GSH synthesis [103]. Cys serves as the initial organic product of sulfur assimilation in plants, and thus, its availability plays a crucial role in determining GSH production [103,104]. Consequently, several regulatory mechanisms influence the GSH biosynthesis process. Various environmental conditions and stresses impact the coarse control of *GSH1* and *GSH2* expression. Additionally, precursor availability affects the system’s activity, and a redox mechanism that considers feedback information finely regulates GSH1 activity. Currently, it remains uncertain whether a redox-coupling mechanism is necessary for the transformation of GSH1 from its oxidized to reduced form. The redox state of GSH, along with its cellular concentration, plays a critical role in mediating and finely regulating metabolism and stress responses. The flavoenzyme NADPH-dependent GSH reductase (GR) efficiently regenerates GSSG to GSH and permits recurrent redox cycling. In order to sustain fundamental functions in the cytosol, mitochondria, and chloroplast stroma in the light, GR contributes to the maintenance of a negative EGSH (i.e., high GSH/GSSG). Gill et al. 2013 [105] conducted a thorough literature analysis about *GR* genes and found evidence that a variety of abiotic stressors, including exposure to HMs, including Cd, salt, drought, UV radiation, and cold temperatures, boost GR activity in many plant species. Moreover, several stress-tolerant plants exhibit significant GR activity [106]. Because of its effective ROS scavenging capacity, transgenic techniques to control GR activity in plants also demonstrate that increased GR activity significantly improves tolerance to oxidative stress brought on by a range of abiotic stimuli [105]. Two nuclear genes, *GR1* (*At3g24170*) and *GR2* (*At3g54660*), which both code for differently localized isoforms, are responsible for encoding GRs in *A. thaliana*. *GR1* is more highly expressed in roots, while *GR2* expression is more pronounced in photosynthetic tissues [98]. *GR1* is localized in the cytoplasm, nucleus, and peroxisomes [107,108,109,110] while *GR2* targets mitochondria as well as chloroplasts [108].

## 5. Transporters Involved in Cd Remediation

There are various forms of Cd in soil, but many of them are not absorbed by plants [111]. For Cd to be absorbed, it must be available for uptake, which depends on the plant species, the physicochemical conditions of the soil, and the speciation of the metals [112,113].

A number of technologies have been developed to lower soil Cd levels. Phytoremediation, used by plants to absorb Cd and store it in easily harvested aerial sections of the plant, is one of the approaches [114]. The choice of appropriate plants that can grow in Cd-polluted soils and accumulate Cd in aerial parts is crucial to the effectiveness of phytoremediation. Over the past ten years, Cd hyperaccumulating plants have received attention [114]. However, only a few plant species that can withstand high amounts of Cd have been identified, including *A. halleri*, *N. caerulescens*, and *S. alfredii.*

Meighan et al., 2011 [115] found that herbaceous Cd hyperaccumulators had limited relevance for phytoremediation in Cd-polluted fields due to their slow development and low biomass output. The use of *Populus* species has been suggested as an alternative for phytoremediation because these woody species can be managed in short rotation coppicing plantations and are known for their fast growth and deep rooting systems [116,117,118,119].

Metal uptake is significantly influenced by root features such as surface area, size, and shape [113,120,121,122,123,124]. Furthermore, plants with thin, hairy roots have demonstrated high absorption and accumulation of metals [125]. In most cases, bivalent forms of trace elements are absorbed [126]. The same transporters that carry Ca, Fe, Mg, Cu, and Zn must carry Cd across root cell walls [15]. By passive transport (diffusion), Cd can move from the soil to plant roots through the cell walls [120,127]. Furthermore, studies have shown that Cd uses nonspecific membrane transport proteins (IRTs and ZIPs) and metal pumping ATPases to facilitate active transport across the plasma membrane of root cells [128,129]. Different transporters are used by plants to sequester metal ions inside organelles, whether in a free ionic state or as chelates. Plants can store extra metals in vacuoles when they are in neutral chelated forms. Long-distance transportation also makes use of chelated metal forms. To lessen the harmful effects of metals, metal chelators can be proteins that bind to metals or other compounds, such as amino acids, their derivatives, or organic acids [130].

These metal chelators also exhibit antioxidant properties. By detoxifying metal ions, specific protein metal chelators like glutaredoxins (GRXs), metallothionins (MTs), and PC_n_ are essential in reducing the effects of metal stress-mediated oxidative damage [130]. Thereby, according to Kumar et al., 2020 [131], the overexpression of glutaredoxin *CaGRX* in chickpeas decreases the absorption of metal ions and boosts the ROS scavenging mechanism, thus mitigating metal stress.

Transporters serve dual roles as receptors and transporters, known as transceptors, within the plant’s sensory apparatus aimed at preserving metal homeostasis [132,133,134,135]. Transceptors have the capability to discern the availability of metal ions in soil and their scarcity within the cytosol, thereby orchestrating the regulation of metal ion uptake through low- or high-affinity transporters. This intricate process leads to a substantial decrease in metal ions, effectively maintaining metal homeostasis within plant cells through the expulsion and exclusion mechanisms facilitated by transporters.

Among these transporters, Cd, Co, Fe, Mn, and Zn divalent ions are transported by the IRT1. Within root cells, IRT1 acts as a transceptor within sensing and signaling mechanisms, prioritizing the sensing of Fe above other ions. Additionally, the IRT1 regulatory domain senses additional ions such as Co, Mn, and Zn, thereby inhibiting IRT1 function [132]. In a different study, CBL-interacting protein kinase 23 (CIPK23) phosphorylates the metal-bound IRT1, leading to ubiquitination by the IRT1 degradation factor 1 (IDF1) protein. This ubiquitination targets IRT1 for breakdown in the vacuole, effectively halting further metal absorption [136].

Wu et al., 2020 [137] discovered in their intriguing study on *A. thaliana* that the use of hydrogen-rich water accelerates the uptake of Cd. Elevated levels of Cd result in hydrogen production, which in turn triggers a cytosolic influx of Ca. Moreover, the activation of respiratory burst oxidase homolog protein D (RBOHD) by Ca elevation results in the production of ROS, primarily H_2_O_2_. In the end, the induced-H_2_O_2_ can inhibit IRT1 transcripts, which will lessen the absorption of more Cd [137].

Another instance in which Cd stress is mitigated is shown in apples, where the kinase salt overly sensitive 2-like1 (SOS2L1) phosphorylates the malate transporter aluminum-activated malate transporter 14 (ALMT14), enhancing its functionality [49]. Otherwise, a rice natural resistance-associated macrophage protein 1 (OsNRAMP1) plasma-membrane-localized Fe transporter that is involved in absorbing Cd is overexpressed, increasing the amount of Cd stored in leaves. Additionally, it was found that the expression of *OsNRAMP1* is correlated with the amount of Cd found across different rice cultivars. Compared to the hyperaccumulating cultivar Sasanishiki, it was expressed up to two times greater in the Cd hyperaccumulating rice cultivar Habataki [138]. According to Lang et al., 2011 [139], *B. juncea* exhibits Cd tolerance through the involvement of two cation efflux transporters, BjCET3 and BjCET4, whose overexpression results in increased Cd accumulation in the shoot without lowering plant biomass.

Otherwise, in *A. thaliana*, the gene *AtNRAMP6* confers Cd tolerance by locating at the Golgi apparatus. By transferring the Fe contained in Golgi vesicles into the cytosol during Fe depletion, *AtNRAMP6* overexpression plays a critical role in preserving intracellular Fe equilibrium [140]. Consequently, by compartmentalizing and redistributing metals to specific organelles, including vacuoles, the endoplasmic reticulum, chloroplasts, and mitochondria, Golgi apparatus-localized metal transporters are necessary to maintain metal homeostasis of Fe, Cd, and Mn.

Furthermore, plants utilize energy-dependent metal transport systems to expel or remove metals, primarily relying on HM ATPases (HMAs) and ABC (ATP-binding cassette) transporters. Typically, these transporters expel metals from root cells into the surrounding air or other sections of the plant, where they can accumulate harmlessly. The role of ABC transporters in the translocation and redistribution of metals has been extensively studied. Fu et al. 2019 [141] demonstrated that Cd treatment in rice promoted the expression of the G-type ABC transporter ABCG36. A loss-of-function mutant exhibited a higher Cd concentration in its roots, suggesting that ABCG36 facilitates Cd translocation from roots to shoots. An interesting study revealed that the *P. tomentosa ABCG36* gene can be overexpressed to improve Cd tolerance in *A. thaliana* plants, suggesting that this transporter serves as a Cd extrusion pump [142]. In accordance with a different study, the *A. thaliana* ABC transporter pleiotropic drug resistance 8 (AtPDR8) acts as a plasma membrane-localized Cd efflux pump, pushing Cd out of the cytosol and onto the cell’s exterior, thus providing HM stress tolerance [143]. In certain plants, only C-type ABC transporters (ABCCs) localize on tonoplasts and contribute to metal tolerance. According to Brunetti et al. 2015 [144] and Song et al. 2010 [145], tonoplast-localized ABCCs in *A. thaliana*, such as AtABCC1, AtABCC2, and AtABCC3, facilitate the transport of PC_n_ and its complexes with As, Cd, Mn, and Zn into the vacuole, thus enhancing metal tolerance.

A recent study compared wild-type (WT) *P. alba* clone Villafranca plants with trans-genic plants overexpressing aquaporin (*aqua1*) in a hydroponic treatment with 10μM Cd. *PaHMA2*, *PaNRAMP1.3*, *PaNRAMP2*, *PaNRAMP3.1*, *PaNRAMP3.2*, *PaABCC9*, and *PaABCC13* gene expressions were evaluated, revealing that the majority of the genes analyzed exhibited a prompt transcriptional response in WT plants at 1 day post-treatment and adaptation at 60 days. Conversely, *aqua1* plants showed a weaker transcriptional response along with a higher Cd content in the medial leaves. In conclusion, *aqua1* overexpression in poplar enhanced Cd translocation, indicating that *aqua1* plants are less sensitive to Cd. Given the significance of *PaNRAMP3* in Cd compartmentalization, a different transcription of this gene in the WT line may account for this distinct response. Nevertheless, even after two months of treatment, Cd administration did not cause observable toxicity symptoms in WT and *aqua1* plants, demonstrating the remarkable resistance of this poplar species to Cd [146].

As part of a redistribution strategy, the HMA family of P1B-ATPases also serves as metal pumps, transporting metals into the xylem, phloem, and apoplast for transfer to other plant components, including shoots and grains. Members of the HMA family are essential for the mobilization of metal ions in a variety of plants, including Cu, Cd, and Zn. HvHMA1 is essential for scavenging Cu and Zn from the chloroplast to the cytosol in barley, facilitating their transport to areas of need [147]. To prevent their intracellular accumulation, HMA2 in *A. thaliana* expels excess cytosolic Zn, Cd, and other ions into the apoplast [148]. In response to Zn deficiency in the shoot, HMA2 and metal transporter protein 2 (MTP2) are stimulated to redistribute Zn from the root to the shoot [149]. Additionally, when cultivated in environments with high concentrations of metals, the HMA2 mutant exhibits raised intracellular Cd and Zn levels.

AtHMA3, localized in tonoplasts, plays a role in storing Cd, Zn, Co, and Pb. According to Morel et al. 2009 [150], overexpression of *AtHMA3* enhances resistance to metal stress and boosts Cd storage capacity. Furthermore, research by Sasaki et al. 2014 [151] observed that vacuolar sequestration of Cd in rice roots contributes to its accumulation. In a comparable way, *GmHMA3* in soybeans is essential for Zn and Cd tolerance. Moreover, a study on rice showed how it has the capability to diminish the quantity of Cd present in its grains by adjusting the Zn-to-Cd ratio via the mutation of OsHMA2 [152].

Additionally, the endomembrane system includes the endoplasmic reticulum (ER), housing a spacious lumen utilized for the storage and utilization of metals for various purposes [153]. Within the ER membrane, there exist broad-specificity transporters for Cd, Cu, and Zn, which are occasionally observed to also localize on the plasma membrane. The endomembrane system’s continuity as an element of the secretory system may be the cause of this shared location. For example, OsZIP1 in rice is situated at both the ER and plasma membrane, primarily in roots where Cd, Cu, and zinc levels are elevated. All the different aforementioned transporters are summarized in Table 1.

## 6. Conclusions

HMs pose significant risks to plant health, causing toxicity, growth inhibition, and oxidative stress. Essential metals are necessary but can be harmful in excess, while non-essential metals like Cd disrupt critical functions. Plants employ various defense mechanisms, including the synthesis of PC_n_ from GSH via PCS, which chelates Cd and facilitates its detoxification by sequestering it into vacuoles. Due to the implication of GSH in mitigating Cd stress, this review highlights the GSH role in response to abiotic stress.

Moreover, plant cells employ a variety of transporters to preserve metal homeostasis. Different transporters are used by plants to sequester metal ions inside organelles, whether in a free ionic state or as chelates. Transporters belonging to the IRT, NRAMP, and HMA subfamily play crucial roles in Cd absorption, translocation, and sequestration across various plant species; ATPases and ABC transporters facilitate the removal of metals from root cells, ensuring metal stress tolerance. Furthermore, the endomembrane system, particularly the ER, houses transporters for Cd, Cu, and Zn, contributing to metal storage and utilization within plant cells.

The limitations of this study include detailing only a few of the methods plants use for detoxification. A more comprehensive study would explore further mechanisms that could act additively or synergistically. It could distinguish mechanisms of avoidance, where the plant limits the entry of metals, such as the release of radical exudates or wall lignification, and mechanisms for damage repair [14,154], in addition to those that have been mentioned in greater depth.

However, this review highlights the importance of understanding the sources and pathways of Cd contamination in soil, emphasizing the need for comprehensive strategies to mitigate HM pollution. By elucidating the physiological, molecular, and biochemical mechanisms underlying plant responses to Cd stress, this work provides a basis for the development of effective strategies to safeguard plant health and ensure ecosystem resilience in the face of HM pollution.

## Figures and Tables

**Table 1:** Main features of transporters implicated in Cd tolerance.

Subfamily	Name	Subcellular Localization	Substrate
IRT	IRT1	Plasma membrane	Fe, Co, Mn, Zn, Cd
SOS	SOS2L1 *	Plasma membrane	Malate and malic acid
ALMT	ALMT14 *	Plasma membrane	Malate and malic acid
CET	BjCET3	Plasma membrane	Zn, Co, Ni, Cd
	BjCET4		
RAMP	OsNRAMP1	Plasma membrane	Fe, Cd
	AtNRAMP6	Golgi	Fe, Mn, Cd
ABCG (PDR)	PtABCG36	Plasma membrane	Pb, Cd
	AtPDR8		
ABCC	AtABCC1	Tonoplast	PC_n_ and its complexes with As, Mn, Zn, Cd
	AtABCC2		
	AtABCC3		
HMA	HvHMA1	Chloroplast	Zn, Cd
	PaHMA2	Plasma membrane	
	AtHMA2		Zn, Cd
	AtHMA3	Tonoplast	Zn, Co, Pb, Cd
ZIP	OsZIP1	Endoplasmic reticulum and plasma membrane	Zn, Cu, Cd

* Not directly involved in Cd transport. Cd presence enhances their functionality, increasing Cd tolerance.

## Data Availability

The data provided in the current review are available from the corresponding author on request.
